# A 2D Photo Score for Phenotypic Severity in Unilateral Coronal Synostosis Patients

**DOI:** 10.1097/SCS.0000000000012989

**Published:** 2026-05-29

**Authors:** Meike C. Tjaberinga, ERN CRANIO–Craniosynostosis Workgroup, I.M.J. Mathijssen

**Affiliations:** Department of Plastic and Reconstructive Surgery, Erasmus University Medical Center, Rotterdam, The Netherlands

**Keywords:** 2D photo score, esthetic outcome assessment, phenotypic severity, suture-specific scoring, unilateral coronal synostosis

## Abstract

**Background:**

Unilateral coronal synostosis (UCS) presents with a wide range of phenotypic severity, yet commonly used outcome measures are heterogeneous and often subjective, resource-intensive, or non–suture-specific. Reliable grading of phenotypic severity is crucial for evaluating aesthetic outcomes and for benchmarking treatment results across centers. The aim of this study was to develop a simple, suture-specific, 2D photo-based scoring system for UCS and to assess its reliability across multiple expert centers.

**Methods:**

A retrospective multicenter study was conducted using standardized photos of patients with nonsyndromic UCS from 6 European expert centers. A UCS-specific photo score was developed through expert consensus and included 5 items: forehead shape, temporal hollowing, orbital dystopia, and nasal deviation, together with an overall phenotype item. All items were scored on a 4-point ordinal scale. A cumulative severity score was calculated from the 4 feature-specific items, excluding the overall phenotype. Twenty craniofacial plastic surgeons and pediatric neurosurgeons independently scored 60 photo sets.

**Results:**

Agreement for individual items was moderate (κ=0.48–0.56), with lower reliability for the overall phenotype item (κ=0.41). The agreement was highest for the cumulative score of feature-specific items (intraclass correlation coefficient: 0.64). Reliability was consistent between 2D photos and 3D-renderings.

**Conclusion:**

This study introduces the first multicenter, suture-specific photo score for UCS. Combining multiple UCS-specific features into a cumulative score substantially improves reliability, supporting its use as a practical tool for standardized phenotypic severity assessment and multicenter benchmarking.

Unilateral coronal synostosis (UCS) is characterized by premature fusion of a single coronal suture, resulting in anterior plagiocephaly with forehead flattening, supraorbital rim retrusion, orbital dystopia, nasal root deviation, and temporal hollowing.^1,2^ The phenotypic severity of UCS varies significantly, ranging from mild to severe cases.^3^ Surgical treatment aims primarily to correct the existing craniofacial asymmetry and improve the conditions for normal, symmetric facial growth.

Accurate assessment of phenotypic severity is essential for evaluating surgical outcomes. It enables comparison across patients, techniques, and treatment centers. Existing approaches range from subjective surgeon impressions and nonstandardized photo review to anthropometric measurements and imaging-based metrics.^4–9^ These latter methods can be time-consuming, demand specialized software, or entail radiation exposure. More advanced 3‑dimensional (3D) morphometric techniques provide objective and spatially detailed shape analysis but require dedicated hardware, software, and expertise, limiting their current use in routine practice.^3,10,11^ Structured 2-dimensional (2D) photo scores offer a low-cost, noninvasive alternative that can be readily integrated into routine clinical practice.

Previously, suture-specific photo scores for sagittal and metopic synostosis have been developed within the European Reference Network for Craniofacial Anomalies (ERN-Cranio) to standardize phenotypic assessment in these types of craniosynostosis.^12,13^ These 2D photo scores enable the structured evaluation of characteristic morphologic features to facilitate comparison across centers. At the time of this writing, a similar photo score for UCS was lacking. Therefore, the aim of this study was to develop a suture-specific photo score for UCS and to evaluate its interrater reliability among expert craniofacial surgeons and pediatric neurosurgeons using 2D photo sets.

## METHODS

### Study Design and Subjects

A retrospective study was conducted using 2D photos of patients with nonsyndromic UCS. Photo sets were eligible if the patients were younger than 18 years and if standardized images were available in 4 views: anterior, right and left lateral, and vertex. Both preoperative and postoperative photo sets were included and were randomly ordered for scoring. When multiple photo sets from the same patient were available, only those taken >5 years apart were retained to minimize the likelihood of recognition.

Six ERN-Cranio expert centers contributed photo sets: Birmingham Children’s Hospital (Birmingham, United Kingdom), Charité-Universitätsmedizin Berlin (Berlin, Germany), Erasmus Medical Center–Sophia Children’s Hospital (Rotterdam, the Netherlands), Fondazione IRCCS Istituto Neurologico Carlo Besta (Milan, Italy), Hospital 12 de Octubre (Madrid, Spain), Vall d’Hebron University Hospital (Barcelona, Spain). Images were provided either as standard color photos or as 2D renderings derived from 3D photogrammetry, depending on local imaging protocols. Photos were screened for adequate visualization of craniofacial features. Images with hair obstruction, excessive facial expression, or parental hands in view were excluded.

### Photo Score Development and Assessment

The development of previous ERN-Cranio photo scores has been detailed previously.^12,13^ Using the same consensus process within the craniosynostosis workgroup, a suture-specific UCS photo score was constructed. Consensus was reached on a suture-specific photo score comprising 5 items, including 4 characteristic features of UCS: forehead shape, temporal hollowing, orbital dystopia, and nasal deviation, together with an overall phenotype item (Fig. 1; Table 1, Supplemental Digital Content 1, http://links.lww.com/SCS/J643). Phenotypic severity for each item was scored on a 4-point ordinal scale, with 0 indicating the absence of the described feature, 1 indicating a mild deformity, 2 indicating moderate deformity, and 3 indicating a severe deformity. A cumulative severity score was calculated from the 4 feature-specific items, excluding the overall phenotype, yielding a possible range of 0 to 12.

**FIGURE 1:**
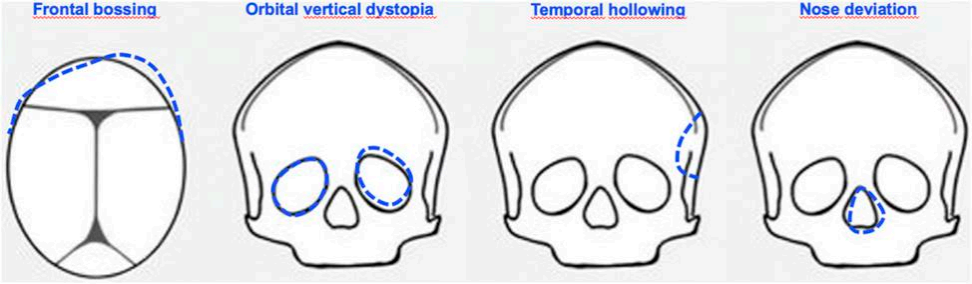
Feature-specific photo score items. Schematic figure of phenotypical features included in the unilateral coronal synostosis photo score, indicated by the blue dotted lines.

An expert panel of craniofacial plastic surgeons and pediatric neurosurgeons within the ERN-workgroup applied the score to all included photo sets. To qualify as a member of the craniosynostosis workgroup, centers must perform at least 20 intracranial procedures for nonsyndromic UCS annually, maintain a multidisciplinary team, and hold national recognition as a referral center. All raters first reviewed 2 practice photo sets to familiarize themselves with the scoring criteria. The practice results were discussed collectively to clarify any remaining ambiguities in item interpretation. Subsequently, all photo sets were scored independently. Images were presented through Microsoft Teams, and scores were collected using Mentimeter. Raters were blinded to patient identity, center of origin, and surgical status. No discussion was allowed during scoring to preserve independent judgment.

### Statistical Analyses

The interrater reliability of the cohort was assessed using a modified weighted Fleiss κ. Ninety-five percent CIs were derived from percentile bootstrapping with 10,000 iterations.^14^ In addition, a cumulative severity score was calculated by adding the scores of all feature-specific items, excluding the overall phenotype item. Interrater reliability of this cumulative score (range: 0–12) was assessed with intraclass correlation coefficients (ICCs) from 2-way random-effects models for single measurements. Both agreement-type and consistency-type ICCs were reported, with CIs obtained using parametric bootstrapping. Variability in agreement between individual raters was explored using pairwise weighted Cohen κ values calculated for each photo score item. The Landis and Koch scale was used to interpret the derived κ values, and, in line with common practice, these thresholds were applied pragmatically to aid interpretation of ICCs.^15^ To assess construct validity, associations between each feature-specific item and overall phenotype severity were examined using the Spearman rank correlation coefficients (*r*_s_), computed per rater and summarized as ranges and medians. In a secondary analysis, interrater reliability was examined by imaging modality (2D photos versus 3D-derived renderings). Statistical analyses were performed in R version 4.4.1.

## RESULTS

### Interrater Reliability

Twenty raters from 16 ERN-Cranio expert centers completed the scoring of all 60 photo sets. The distribution of item-specific severity scores is shown in Figure 2. Interrater reliability for each individual item and the ICC of the cumulative score (excluding “overall phenotype”) are presented in Table 2A, Supplemental Digital Content 1, http://links.lww.com/SCS/J643. The interrater reliability showed moderate agreement among raters for all individual items. Temporal hollowing demonstrated the highest agreement, with a modified Fleiss κ of 0.56 (95% CI: 0.53–0.58). Forehead shape and nose deviation showed comparable levels of agreement (0.53, 95% CI: 0.50–0.56 and 0.51, 95% CI: 0.47–0.55, respectively). Orbital dystopia showed slightly lower but still moderate agreement, with a coefficient of 0.48 (95% CI: 0.44–0.52). The overall phenotype item yielded the lowest reliability among the assessed features, with a modified Fleiss κ of 0.41 (95% CI: 0.37–0.44). All items demonstrated agreement significantly above chance.

**FIGURE 2:**
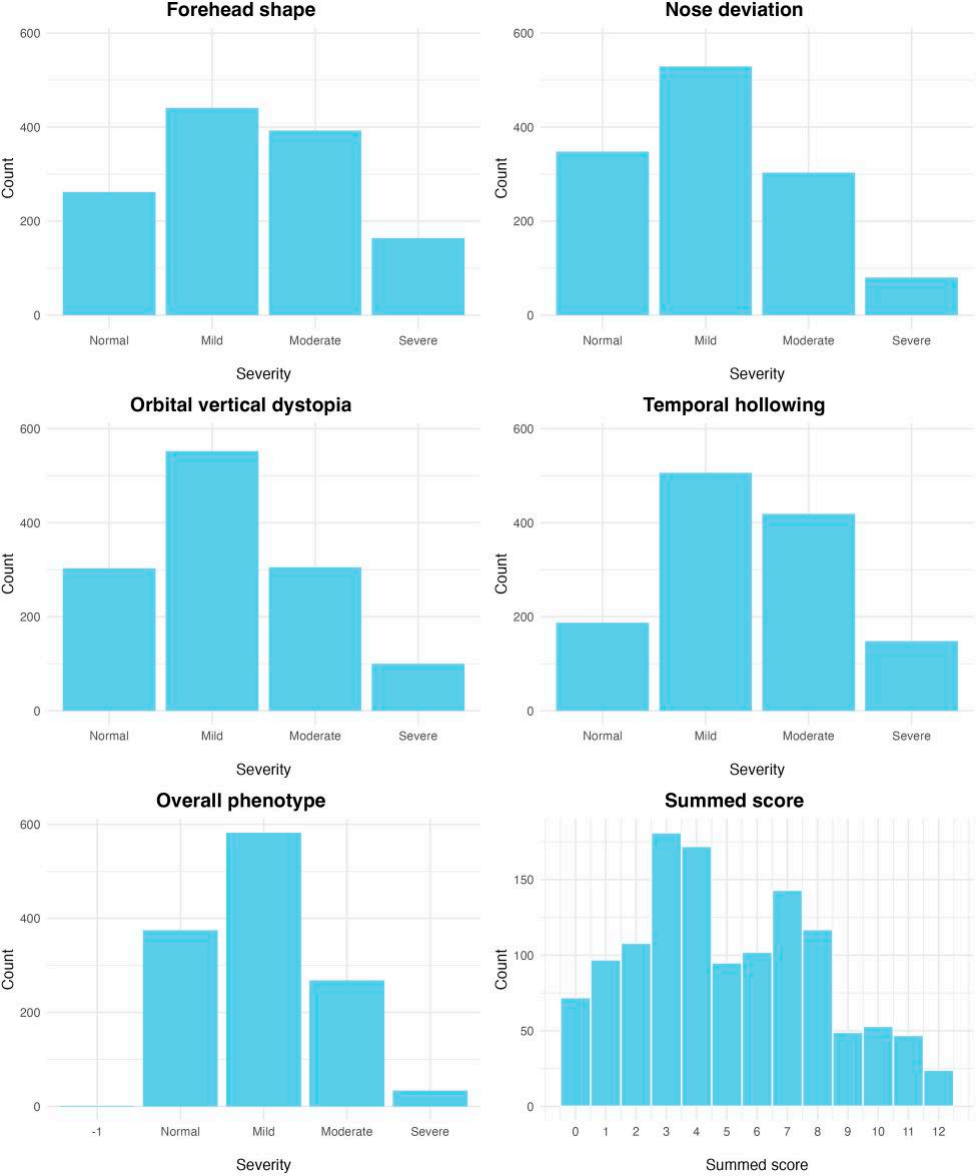
Total scores for each photo score item and the summed score.

When the 4 feature-specific items were combined into a cumulative severity score, interrater reliability increased substantially. The agreement-type ICC was 0.66 (95% CI: 0.57–0.74), and the consistency-type ICC was 0.71 (95% CI: 0.63–0.78), indicating substantial agreement among raters (Table 2B, Supplemental Digital Content 1, http://links.lww.com/SCS/J643).

Pairwise weighted κ analyses demonstrated substantial variability in agreement across raters (Fig. 3). The 4 feature-specific items showed κ values ranging from slight to moderate agreement, with forehead shape (κ min=0.16, κ max=0.65), nose deviation (κ min=0.07, κ max=0.59), orbital dystopia (κ min=0.04, κ max=0.59), and temporal hollowing (κ min=0.09, κ max=0.69). The overall phenotype item displayed the widest spread in agreement across the scoring panel (κ min=−0.24, κ max=0.48).

**FIGURE 3:**
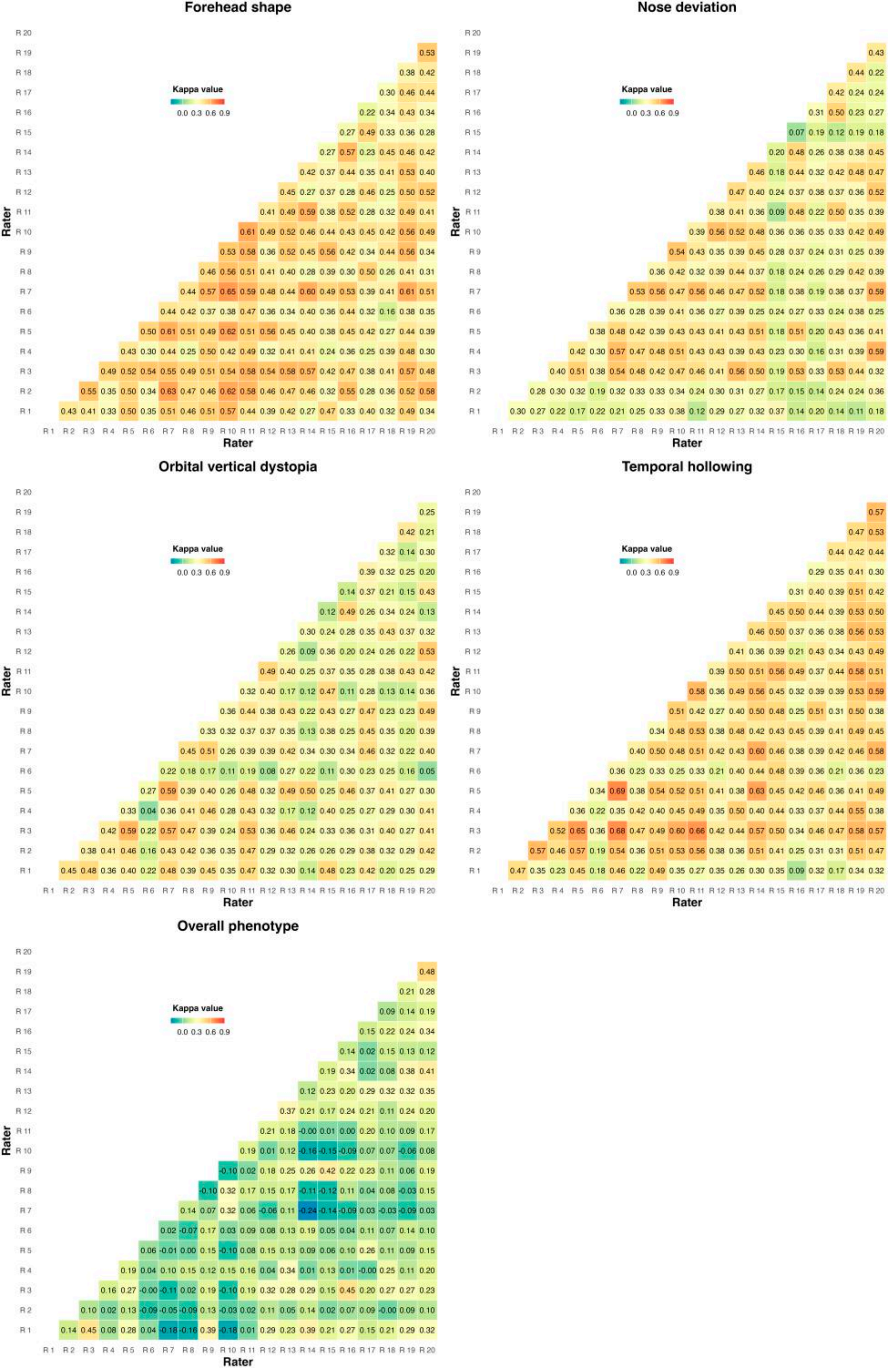
Pairwise κ for all photo score items.

### Relation Between Subitems and Overall Phenotype Assessment

The strength of the association between individual items and the overall phenotype varied considerably across raters (Fig. 4). Temporal hollowing demonstrated the strongest association with the overall phenotype (*r*_s_ range: –0.20 to 0.93; median: 0.53), followed by forehead shape (*r*_s_ range: –0.40 to 0.88; median: 0.43). Orbital dystopia and nose deviation showed weaker and more heterogeneous associations (orbital dystopia: *r*_s_ range: –0.23 to 0.77; median: 0.35; nose deviation: *r*_s_ range, –0.37 to 0.83; median: 0.30). Across all items, *r*_s_ values spanned from strongly negative to strongly positive, illustrating a broad range in correlations between individual feature scores and the overall phenotype across raters (Table 3, Supplemental Digital Content 1, http://links.lww.com/SCS/J643).

**FIGURE 4:**
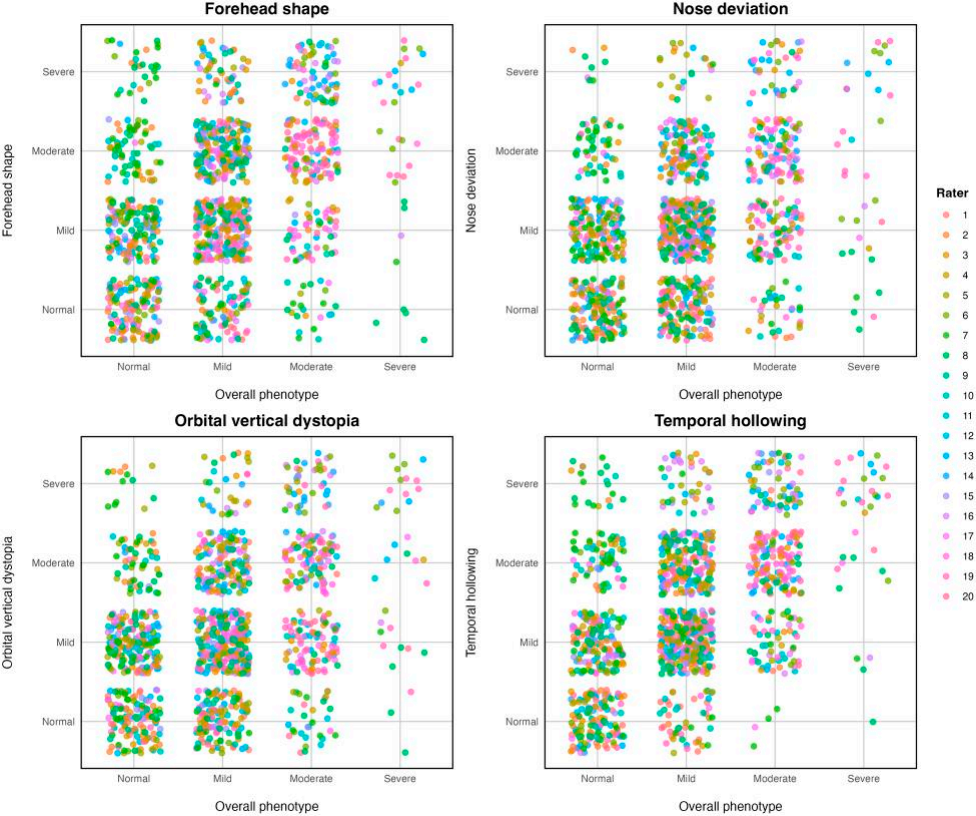
Correlation between individual photo-score items and the overall phenotype.

### Reliability by Imaging Modality

Interrater reliability estimates for 3D-derived photo renderings and conventional 2D photos were of similar magnitude, although CIs were wide for the 3D-derived subgroup. For the individual items, modified Fleiss κ values for 3D-derived images ranged from 0.39 for the overall phenotype to 0.59 for temporal hollowing, whereas κ values for 2D photos ranged from 0.41 to 0.55 across the same items. For the cumulative score, the agreement-type ICC was 0.66 (95% CI: 0.43–0.91) for 3D-derived images and 0.64 (95% CI: 0.55–0.74) for 2D photos, with corresponding consistency-type ICCs of 0.76 (95% CI: 0.55–0.94) and 0.69 (95% CI: 0.61–0.77), respectively (Table 4, Supplemental Digital Content 1, http://links.lww.com/SCS/J643).

## DISCUSSION

This study aimed to develop and evaluate a simple, suture-specific photo score to determine the phenotypic severity of UCS. In a multicenter panel of experienced craniofacial and pediatric neurosurgeons, agreement for the individual items was moderate, whereas the cumulative severity score showed substantial interrater reliability. The score performed similarly across centers and when applied to both 2D photos and 3D-derived renderings. To our knowledge, this score represents the first UCS instrument tested for interrater reliability across multiple expert centers. As such, it is intended for standardized severity grading in routine practice and for use as a common esthetic outcome measure in registries and future multicenter studies.

Several studies have investigated clinical severity and esthetic outcomes in UCS. Widely used systems, such as the Whitaker classification, emphasize the need for revision surgery rather than phenotypic severity and lack suture specificity, limiting their usefulness for detailed esthetic evaluation and benchmarking.^16–18^ Quantitative approaches, including anthropometric measurements, cephalometrics, and CT-based indices, describe UCS-related deformity in detail but are less suited to routine, longitudinal, and large-scale comparison across centers.^4,5,7,9,11^ To date, only 1 previous study has used a 2D photo score specifically designed to assess phenotypic severity in UCS patients. In a single-center study, a junior plastic surgeon and a medical student scored forehead shape, orbital dystopia, and temporal depression on a 3-point scale and reported moderate to almost perfect agreement for individual items (κ=0.65–0.82).^19^ The higher agreement on the individual items, compared with the present study, likely reflects a smaller, more homogeneous rater panel, use of a simpler rating scale with a neutral midpoint, and local calibration of feature interpretation. Although agreement for individual items was moderate in the multicenter expert panel, reliability improved when multiple UCS-specific features were combined in the cumulative score.

A notable observation in this study was the limited use of the “severe” category in the overall phenotype rating. This likely reflects both the composition of the data set, in which half of the images were postoperative, and rater conservatism in assigning the highest severity category. As a result, overall phenotype scores clustered toward the lower end of the scale, leading to a compressed distribution with reduced discriminatory capacity. Such restriction of range limits achievable correlations with individual items and may partly explain the heterogeneous item-overall phenotype associations observed across raters. In contrast, the cumulative severity score demonstrated substantially higher interrater reliability, with ICC values in the substantial range. By integrating multiple feature-specific items, the cumulative score mitigates the impact of rater-specific weighting and scale compression, providing a more stable and reproducible measure of phenotypic severity. These findings support the cumulative score as the preferred metric for future applications of this photo score, particularly in multicenter settings and studies combining pre- and postoperative images.

The UCS photo score aligns closely with the previously established sagittal and metopic photo scores in its overall design and performance. Although the specific items differ by synostosis type, all scores include assessment of forehead shape and temporal hollowing.^12,13^ In contrast to sagittal and metopic assessments, the UCS score focuses on asymmetry of craniofacial features rather than global deviation of skull shape from normal. These phenotypic differences are reflected in the reliability estimates. For the overall phenotype item, agreement in UCS was in the same moderate range but presented a lower (Fleiss κ=0.41) than in sagittal and metopic synostosis (0.53 and 0.56, respectively). This likely reflects an inherent limitation of 2D photography for capturing a fundamentally 3D, asymmetric deformity in UCS. In contrast, 2D photographs are better suited to capture the predominantly symmetric shape changes seen in scaphocephaly and trigonocephaly. Nevertheless, similar to the sagittal and metopic scores, the cumulative UCS severity score still reached substantial reliability. This suggests that aggregating multiple items can partially compensate for lower agreement on individual features.

Across imaging modalities, performance of the UCS photo score was consistent, supporting its use for benchmarking within collaborative networks. Reliability estimates were similar for 2D photos and 3D‑derived images. Because subgroup sizes were small and CIs were wide, the stratified analyses warrant cautious interpretation. Nonetheless, the overall findings indicate that the cumulative severity score can be applied across centers with different infrastructures and 2D/3D workflows.

This study has several limitations that should be considered when interpreting the findings. First, the UCS photo score remains an expert-based visual rating tool and therefore retains an inherently subjective component. To minimize this subjectivity, predefined items with explicit severity descriptors were used, and interrater agreement was formally assessed. Second, unlike true morphometric measurements, 2D photos do not capture full 3D craniofacial shape. Residual variation in image acquisition, including head position, facial expression, lighting, and distance and angle of the camera, may have influenced scoring, despite efforts to standardize photographic protocols. Third, the photo score was intentionally developed for use by expert craniofacial surgeons and pediatric neurosurgeons within a registry setting. However, differences in clinical experience and case exposure may influence how phenotypic severity is interpreted. Surgeons working in high-volume referral centers may calibrate severity differently from those with limited exposure to severe cases. Short rater training or calibration sessions with shared reference cases may help align these perspectives and reduce this source of variability in future applications.

Recent advances in 3D photogrammetry and surface-based morphometrics have demonstrated the potential of radiation-free, objective, and reproducible quantification of craniofacial form and severity.^11,20–23^ Emerging artificial-intelligence and deep-learning-based approaches further expand these possibilities by enabling automated landmark recognition, objective asymmetry quantification, and standardized frameworks for evaluating craniofacial shape.^24,25^ However, variability in analytic pipelines, limited external validation, and the absence of universally accepted outcome definitions at the time of this writing limit their use for multicenter benchmarking and routine clinical implementation.^26,27^ ​ In this evolving landscape, the UCS photo score should be viewed as a pragmatic and complementary tool. It enables standardized phenotypic assessment and multicenter benchmarking in large cohorts using routinely available 2D and 3D-derived images, while a consensus on 3D analytic methods is still evolving. Future work should integrate UCS photo score outcomes with standardized 3D morphometric metrics and patient-reported outcome measures to better connect subjective esthetic assessment with objective characterization of craniofacial shape and patient experience.

## CONCLUSION

This multicenter study evaluated a suture-specific photo score for quantifying phenotypic severity in nonsyndromic UCS. Severity ratings for individual craniofacial features showed only moderate agreement, whereas the cumulative severity score achieved substantial reliability, indicating greater concordance when multiple items are combined into an overall assessment. These findings support the UCS photo score as a pragmatic tool for standardized phenotypic severity grading and underscore the need for complementary objective outcome measures in future benchmarking studies.

## ERN CRANIO—Craniosynostosis Workgroup

## Supplementary Material
