# Supplementary material for: A 2D Photo Score for Phenotypic Severity in Unilateral Coronal Synostosis Patients
Source: J Craniofac Surg. 2026 May 29;37(10):2628–32. doi: 10.1097/SCS.0000000000012989 (PMC13645408; doi:10.1097/SCS.0000000000012989)
Supplement: Supplementary file 1 [file scs-37-02628-s001.docx]

**Supplemental Digital Content 1. Description of the photo score items**

| **Photo score item** | **Description** | **View** |
| --- | --- | --- |
| 1. Forehead shape | Flattening of the forehead on affected side; bulging forehead on the contralateral side | Vertex |
| 2. Orbital dystopia | Elevation of the orbit on the affected side | Anterior |
| 3. Temporal hollowing | Abnormal temporal hollowing above the supraorbital bar on the lateral sides of the affected side | Anterior, lateral |
| 4. Nose deviation | Deviation of the nose towards the affected side | Anterior |
| 5. Overall phenotype | An overall assessment of facial appearance reflecting the global facial symmetry | All |

**Supplemental Digital Content 2**

| **Photo score item** | **Modified Fleiss‘ Kappa statistic** | **95% bootstrap CI** | **Agreement strength** |
| --- | --- | --- | --- |
| Forehead shape | 0.53 | 0.50; 0.56 | Moderate |
| Nose deviation | 0.51 | 0.47; 0.55 | Moderate |
| Orbital dystopia | 0.48 | 0.44; 0.52 | Moderate |
| Temporal hollowing | 0.56 | 0.53; 0.58 | Moderate |
| Overall phenotype | 0.41 | 0.37; 0.44 | Moderate |

**Supplemental Table 2A.** Interrater reliability of each item in the photo score. All items have a moderate agreement strength on Landis and Koch scale.

| **ICC^1^ type** | **ICC** | **95% CI^2^** | **Agreement strength** |
| --- | --- | --- | --- |
| Agreement | 0.66 | 0.57, 0.74 | Substantial |
| Consistency | 0.71 | 0.63, 0.78 | Substantial |

**Supplemental Table 2B.** Intraclass correlation coefficient for summed scores. Both are considered substantial agreement strength on Landis and Koch scale.

^1^ICC = Intraclass correlation coefficient

^2^CI= Confidence interval

**Supplemental Digital Content 3**

| **Item** | **r_s_^1^ min** | **r_s_ max** | **r_s_ median** |
| --- | --- | --- | --- |
| Forehead shape | −0.40 | 0.88 | 0.43 |
| Nose deviation | −0.37 | 0.83 | 0.3 |
| Orbital vertical dystopia | −0.23 | 0.77 | 0.35 |
| Temporal hollowing | −0.20 | 0.93 | 0.53 |

**Supplemental Table 3A.** Correlation between feature specific item scores and overall phenotype

| **Rater** | **Forehead shape r_s_ (95% CI^2^)** | **Nose deviation r_s_ (95% CI)** | **Orbit dystopia r_s_ (95% CI)** | **Temporal hollowing r_s_ (95% CI)** |
| --- | --- | --- | --- | --- |
| R 1 | 0.63 (0.44, 0.77) | 0.29 (0.05, 0.51) | 0.48 (0.26, 0.65) | 0.70 (0.57, 0.81) |
| R 2 | 0.38 (0.17, 0.57) | 0.29 (0.05, 0.52) | 0.25 (0.03, 0.46) | 0.47 (0.27, 0.64) |
| R 3 | 0.60 (0.41, 0.75) | 0.45 (0.23, 0.64) | 0.40 (0.18, 0.59) | 0.59 (0.39, 0.74) |
| R 4 | 0.22 (-0.03, 0.46) | 0.13 (-0.10, 0.34) | 0.19 (-0.06, 0.43) | 0.39 (0.17, 0.57) |
| R 5 | 0.38 (0.14, 0.59) | 0.27 (0.03, 0.48) | 0.18 (-0.08, 0.43) | 0.41 (0.20, 0.60) |
| R 6 | 0.53 (0.32, 0.70) | 0.64 (0.49, 0.76) | 0.63 (0.43, 0.79) | 0.73 (0.59, 0.83) |
| R 7 | -0.40 (-0.61, -0.16) | -0.28 (-0.49, -0.05) | -0.23 (-0.46, 0.02) | -0.20 (-0.42, 0.05) |
| R 8 | -0.27 (-0.50, -0.02) | -0.12 (-0.37, 0.13) | -0.10 (-0.33, 0.14) | -0.02 (-0.28, 0.23) |
| R 9 | 0.56 (0.41, 0.70) | 0.46 (0.25, 0.64) | 0.51 (0.32, 0.66) | 0.64 (0.49, 0.77) |
| R 10 | -0.37 (-0.57, -0.15) | -0.41 (-0.62, -0.17) | -0.03 (-0.27, 0.21) | -0.14 (-0.37, 0.11) |
| R 11 | 0.17 (-0.08, 0.41) | 0.21 (-0.01, 0.41) | 0.01 (-0.25, 0.26) | 0.21 (-0.07, 0.45) |
| R 12 | 0.42 (0.17, 0.63) | 0.31 (0.08, 0.50) | 0.34 (0.11, 0.55) | 0.60 (0.42, 0.73) |
| R 13 | 0.72 (0.57, 0.82) | 0.53 (0.32, 0.70) | 0.61 (0.42, 0.75) | 0.77 (0.63, 0.86) |
| R 14 | 0.80 (0.66, 0.90) | 0.83 (0.72, 0.91) | 0.77 (0.66, 0.86) | 0.82 (0.73, 0.88) |
| R 15 | 0.56 (0.36, 0.72) | 0.25 (0.02, 0.44) | 0.28 (0.00, 0.53) | 0.57 (0.37, 0.71) |
| R 16 | 0.37 (0.13, 0.58) | 0.35 (0.10, 0.57) | 0.33 (0.10, 0.55) | 0.38 (0.12, 0.60) |
| R 17 | 0.42 (0.19, 0.62) | 0.24 (-0.01, 0.48) | 0.38 (0.14, 0.59) | 0.33 (0.08, 0.55) |
| R 18 | 0.43 (0.21, 0.61) | 0.55 (0.37, 0.70) | 0.51 (0.31, 0.69) | 0.48 (0.26, 0.67) |
| R 19 | 0.88 (0.81, 0.94) | 0.54 (0.31, 0.72) | 0.54 (0.32, 0.72) | 0.93 (0.86, 0.97) |
| R 20 | 0.70 (0.56, 0.82) | 0.53 (0.35, 0.67) | 0.35 (0.13, 0.55) | 0.74 (0.62, 0.84) |

**Supplemental Table 3B.** Spearman correlation between item scores and overall phenotype per rater

^1^ r_s_ = Spearman correlation

^2^CI= Confidence interval

**Supplemental Digital Content 3. Interrater reliability for 3D photosets and 2D photosets**

| **Item** | **2D photographs, κ^1^ (95% CI^2^)** | **3D-derived, κ (95% CI)** |
| --- | --- | --- |
| Forehead shape | 0.52 (0.49-0.55) | 0.56 (0.50–0.63) |
| Nose deviation | 0.52 (0.48–0.56) | 0.44 (0.36–0.52) |
| Orbital vertical dystopia | 0.49 (0.45–0.53) | 0.42 (0.32–0.50) |
| Temporal hollowing | 0.55 (0.52–0.58) | 0.59 (0.51–0.68) |
| Overall phenotype | 0.41 (0.37–0.45) | 0.39 (0.30–0.49) |

**Supplemental Table 4A.** Modified Fleiss’ kappa (linear weights) by item for 3D-derived vs 2D photo sets.

| **Photo type** | **ICC^3^ agreement (95% CI)** | **ICC consistency (95% CI)** |
| --- | --- | --- |
| 3D-derived | 0.66 (0.43–0.91) | 0.76 (0.55–0.94) |
| 2D photographs | 0.64 (0.55–0.74) | 0.69 (0.61–0.77) |

**Supplemental Table 4B.** Intraclass correlation coefficients of sum scores for 3D-derived vs 2D photo sets.

^1^κ = Modified Fleiss’ kappa

^2^CI= Confidence interval

^3^ICC = Intraclass correlation coefficient
